# Anisotropy of Anomalous Diffusion Improves the Accuracy of Differentiating and Grading Alzheimer's Disease Using Novel Fractional Motion Model

**DOI:** 10.3389/fnagi.2020.602510

**Published:** 2020-11-19

**Authors:** Lei Du, Zifang Zhao, Boyan Xu, Wenwen Gao, Xiuxiu Liu, Yue Chen, Yige Wang, Jian Liu, Bing Liu, Shilong Sun, Guolin Ma, Jiahong Gao

**Affiliations:** ^1^Department of Radiology, China-Japan Friendship Hospital, Beijing, China; ^2^Graduate School of Peking Union Medical College, Chinese Academy of Medical Sciences and Peking Union Medical College, Beijing, China; ^3^Department of Anesthesiology, Peking University First Hospital, Peking University, Beijing, China; ^4^Beijing Intelligent Brain Cloud Inc., Beijing, China; ^5^Department of Ultrasound Diagnosis, China-Japan Friendship Hospital, Beijing, China; ^6^Beijing City Key Lab for Medical Physics and Engineering, Institute of Heavy Ion Physics, School of Physics, Peking University, Beijing, China; ^7^Center for MRI Research, Academy for Advanced Interdisciplinary Studies, Peking University, Beijing, China; ^8^McGovern Institute for Brain Research, Peking University, Beijing, China

**Keywords:** diffusion magnetic resonance imaging, fractional motion model, anisotropy, Alzheimer's disease, hippocampus

## Abstract

**Background and Purpose:** Recent evidence shows that the fractional motion (FM) model may be a more appropriate model for describing the complex diffusion process of water in brain tissue and has shown to be beneficial in clinical applications of Alzheimer's disease (AD). However, the FM model averaged the anomalous diffusion parameter values, which omitted the impacts of anisotropy. This study aimed to investigate the potential feasibility of anisotropy of anomalous diffusion using the FM model for distinguishing and grading AD patients.

**Methods:** Twenty-four patients with AD and 11 matched healthy controls were recruited, diffusion MRI was obtained from all participants and analyzed using the FM model. Generalized fractional anisotropy (gFA), an anisotropy metric, was introduced and the gFA values of FM-related parameters, Noah exponent (α) and the Hurst exponent (*H*), were calculated and compared between the healthy group and AD group and between the mild AD group and moderate AD group. The receiver-operating characteristic (ROC) analysis and the multivariate logistic regression analysis were used to assess the diagnostic performances of the anisotropy values and the directionally averaged values.

**Results:** The gFA(α) and gFA(*H*) values of the moderate AD group were higher than those of the mild AD group in left hippocampus. The gFA(α) value of the moderate AD group was significantly higher than that of the healthy control group in both the left and right hippocampus. The gFA(ADC) values of the moderate AD group were significantly lower than those of the mild AD group and healthy control group in the right hippocampus. Compared with the gFA(α), gFA(*H*), α, and *H*, the ROC analysis showed larger areas under the curves for combination of α + gFA(α) and the combination of *H* + gFA(*H*) in differentiating the mild AD and moderate AD groups, and larger area under the curves for combination of α + gFA(α) in differentiating the healthy controls and AD groups.

**Conclusion:** The anisotropy of anomalous diffusion could significantly differentiate and grade patients with AD, and the diagnostic performance was improved when the anisotropy metric was combined with commonly used directionally averaged values. The utility of anisotropic anomalous diffusion may provide novel insights to profoundly understand the neuropathology of AD.

## Introduction

Alzheimer's disease (AD), manifested as progressive cognitive decline and memory loss, is the most common neurodegenerative disease (Reddy and Oliver, 2019). Approximately accounting for 60–70% of dementia patients, AD has been the most prevalent type of dementia (Wortmann, 2012; Alzheimer's, 2016; Khan et al., 2017). The underlying neuropathological mechanisms of AD include the hyperphosphorylation of tau protein and the deposition of β-amyloid (Aβ), which lead to the formation of intracellular neurofibrillary tangles (NFTs) and Aβ plaques separately (Kidd, 1963; Hyman et al., 1984; Braak and Braak, 1991; Wegmann et al., 2010; Mattsson et al., 2019) and ultimately result in the apoptosis of neurons. Neuropathological changes can occur and persist for decades before the appearance of cognitive degeneration. Recently, a variety of magnetic resonance imaging (MRI) techniques have been widely investigated for the diagnosis of AD; however, these methods are insufficient to make a specific diagnosis of AD (Cummings, 2017; Mattsson et al., 2019).

Diffusion MRI (dMRI) can non-invasively describe the random motion of water molecules in and around brain structures such as cell bodies or brain white matter fibers, which provides rich information of microscopic properties than other traditional MRI sequences (Le Bihan, 1995; Le Bihan and Johansen-Berg, 2012; Harrison et al., 2020) and has become a widely used imaging practice in clinical practice and relevant researches (La Rocca et al., 2018; Anckaerts et al., 2019; Bergamino et al., 2020; Finsterwalder et al., 2020). Directional dependence (i.e., anisotropy) is one of the most important microscopic properties obtained from the nervous system by dMRI. Anisotropy results from the dense accumulation of axons and inherent axonal membranes, which prevent the diffusion of water perpendicular to the long axis of fibers (Beaulieu, 2002). One of the most commonly used diffusion MRI technologies, the apparent diffusion coefficient (ADC), was found useful in differentiating AD patients (Takahashi et al., 2017; Xue et al., 2019) and AD transgenic mice (Thiessen et al., 2010). Moreover, the ADC value of white matter in the frontal lobe was correlated with mini-mental state examination (MMSE) scale (Xue et al., 2019). Diffusion tensor imaging (DTI) is another commonly used diffusion MRI technology to measure the anisotropy in the research (Basser et al., 1994a,b). DTI has been increasingly applied to the diagnosis of AD in both basic and clinical studies. The degree of diffusion anisotropy is mostly quantified by two DTI-derived metrics, the fractional anisotropy (FA) and mean diffusivity (MD), in patients with AD (Mayo et al., 2017; Brueggen et al., 2019; Marcos Dolado et al., 2019). Several studies found that the FA values reduced and MD values increased in the hippocampus of AD patients and amnestic mild cognitive impairment (aMCI) patients when compared with healthy control. And the FA and MD might be used to differentiate healthy controls, aMCI patients, and AD patients (Hong et al., 2013; Tang et al., 2016; Schouten et al., 2017). Moreover, the FA value or MD value of hippocampus could be used to predict the progression of AD or aMCI, which is evaluated by MMSE scale (Hong et al., 2013; Lee et al., 2017), indicating the possibility of diffusivity as a biomarker for disease progression. In addition to DTI combined with functional MRI, structural MRI can improve the diagnostic accuracy of AD (Dyrba et al., 2015; Tang et al., 2016; Bouts et al., 2018).

DTI presumes a normal diffusion process in brain tissues and is consequently quantified using a mono-exponential model, *S*/*S*_0_ = exp (–*b* · ADC). The b-value represents the applied magnetic field gradient sequence. However, it has been recognized that the observed dMRI signal decay curve deviates from the mono-exponential form in brain tissues, especially at high b-values (De Santis et al., 2011). To solve this problem, several models have been developed based on different theories of anomalous diffusion processes to find the optimal consistency between the observed signal decay curve and the fitted curves. Representative models include the stretched exponential model (Bennett et al., 2003), the bi-exponential model (Mulkern et al., 1999), the kurtosis model (Jensen et al., 2005), and the statistical model (Yablonskiy et al., 2003). Additionally, several physics-motivated dMRI models have also been proposed (Magin et al., 2008; Ingo et al., 2014).

The fractional motion (FM) model has been proposed as a more appropriate approach to describe the complex diffusion process of biological systems (Magdziarz et al., 2009; Burnecki and Weron, 2010; Weiss, 2013). Theoretically, the FM model presumes that the diffusion process of water molecules is α-stable and *H*-self-similar and has stationary increments. The symbol α represents the Noah exponent, which describes the fluctuations of the random process. The symbol *H* represents the Hurst exponent, which depicts the self-similarity property of molecular trajectories. The FM model possesses a relevantly more excellent consistency between experimental data and fitting curves. Many studies have demonstrated the clinical feasibility of anomalous diffusion using the FM model (Kwee et al., 2010a,b; Sui et al., 2015; Karaman et al., 2016; Xu et al., 2017b, 2018; Du et al., 2020). In the aforementioned studies, researchers averaged the anomalous diffusion parameter values that were acquired in different gradient directions, which ignored the impacts of anisotropy. However, existing literature elucidated that the anisotropy of anomalous diffusion should not be neglected as it revealed a different image contrast and provided unique information (Hall and Barrick, 2012; Xu et al., 2017a). At present, the availability regarding the clinical application of the anisotropy of anomalous diffusion in AD patients remains unclear. The purpose of this study was to investigate the potential feasibility of anisotropy of anomalous diffusion for distinguishing AD patients from healthy controls and grading AD patients.

## Materials and Methods

### Subjects

This research was approved by the ethics committee of the China-Japan Friendship Hospital, and the informed consent was obtained from all subjects. The cognitive function of all participants was assessed by the MMSE scale and Montreal cognitive assessment (MoCA) scale. Initially, MRI examinations were performed on 13 healthy controls and 50 patients with AD. The patients with AD visited the Department of Neurology of the China-Japan Friendship Hospital from November 2015 to March 2019. The clinical diagnosis of AD met the criteria of the National Institute of Neurological and Communicative Disorders and Stroke and the Alzheimer's Disease and Related Disorders Association (NINCDS-ADRDA) (1984) (McKhann et al., 1984; Mattsson et al., 2019). Only the mild-to-moderate AD patients (11 ≤ MMSE score ≤ 25) (Folstein et al., 1975; Perneczky et al., 2006; Tchalla et al., 2018) who met the following criteria were considered for inclusion: (a) the participants' acquired MR image had no artifacts; (b) the participants had no other brain diseases, such as cerebral ischemia or infarction; and (c) the participants had no visual and hearing impairment disorders, aphasia, and limb activity disorder. Finally, 24 patients with AD were eligible and enrolled in this study (9 males and 15 females, mean age, 69.0 years, age range, 50–79 years). Healthy controls were recruited from the local community. Inclusion criteria were as follows: (a) ages range from 50 to 79 years (including 50 and 79 years); (b) a degree of primary education or above; and (c) neurological examination showed no obvious anomalies, and the MMSE scores were between 26 and 30. Healthy controls who suffered from cardiovascular, neurologic, metabolic, and psychiatric disorders or brain abnormalities were excluded. Eventually, 11 healthy controls (2 males and 9 females, mean age 65.3 years, range 54–78 years) were enrolled in the present study. Detailed demographic and clinical characteristics of all participants are summarized in Table 1.

**Table 1:** Demographic and clinical information of all participants.

	**Healthy controls**	**AD patients**		***P*–value**
		**Mild AD**	**Moderate AD**	
Number	11	12	12	-
Male/female	2/9	6/6	3/9	>0.05
Age	65.3 ± 6.6	65.8 ± 10.1	72.1 ± 3.8	>0.05
Education	10.6 ± 3.3	13.4 ± 3.1	10.5 ± 3.9	>0.05
MMSE score	28.8 ± 1.1	23.2 ± 1.3	19.1 ± 1.4	<0.05
MoCA score	-	19.5 ± 2.4	16.5 ± 2.2	<0.05

*The MoCA score was only compared between mild AD group and moderate AD group using a two-sample t-test*.

*AD, Alzheimer's disease; MMSE, mini-mental state examination; MoCA, Montreal cognitive assessment*.

### Image Acquisition

All participants received conventional MRI, 3D T1-weighted imaging, and dMRI. The MRI scans were performed on a 3.0-T MRI scanner (GE Healthcare, Discovery MR750, USA) equipped with an eight-channel head coil. dMR images of all participants were obtained using a special Stejskal–Tanner single-shot spin-echo echo-planar-imaging sequence.

To fit the FM model, we did not fix the diffusion gradient separation time (Δ) during the scanning process as the conventional dMRI sequence. Specifically, Δ was arrayed at 27.060, 39.560, and 52.060 ms. For each Δ value, the diffusion gradient amplitude (G_0_) was 15.67, 19.68, 24.73, 31.06, 39.01, and 49.00 mT/m in sequence, which were selected to be approximately evenly spaced on the log axis. The gradient duration constant (δ) was set to 20.676 ms. Thereafter, 18 non-zero b-values (151, 239, 377, 595, 939, and 1,481 s/mm^2^ for Δ at 27.060 ms; 245, 387, 611, 964, 1,521, and 2,399 s/mm^2^ for Δ at 39.560 ms; and 339, 535, 845, 1,333, 2,103, and 3,317 s/mm^2^ for Δ at 52.060 ms) were obtained in each gradient direction. In order to decrease the effect of diffusion anisotropy, we successively applied the diffusion gradients in three orthogonal directions (the x-axis, y-axis, and z-axis) in turn. Moreover, a total of 12 images without diffusion sensitization (b = 0) were acquired.

The dMRI scanning parameters included the following: repetition time (TR)/echo time (TE) = 3,800 ms/110 ms; accelerating factor = 2; flip angle = 90°; number of excitations = 2; field of view (FOV) = 240 mm × 240 mm; matrix size = 128 × 128; slice thickness = 5.0 mm; number of slices = 27; voxel size = 1.875 × 1.875 × 5 mm^3^. Since high in-plane resolution was preferable, a large slice thickness had to be chosen to achieve a decent signal-to-noise ratio (SNR). The total scan time was 8 min 33 s, which facilitated the clinical use. T1 structure image parameters were as follows: TR = 6.7 ms; TE = min full; flip angle = 12°; FOV = 256 mm × 256 mm; matrix size = 256 × 256; slice thickness = 1.0 mm; number of slices = 192; scan time = 4 min 10 s.

### Image Segmentation

**Figure 1:**
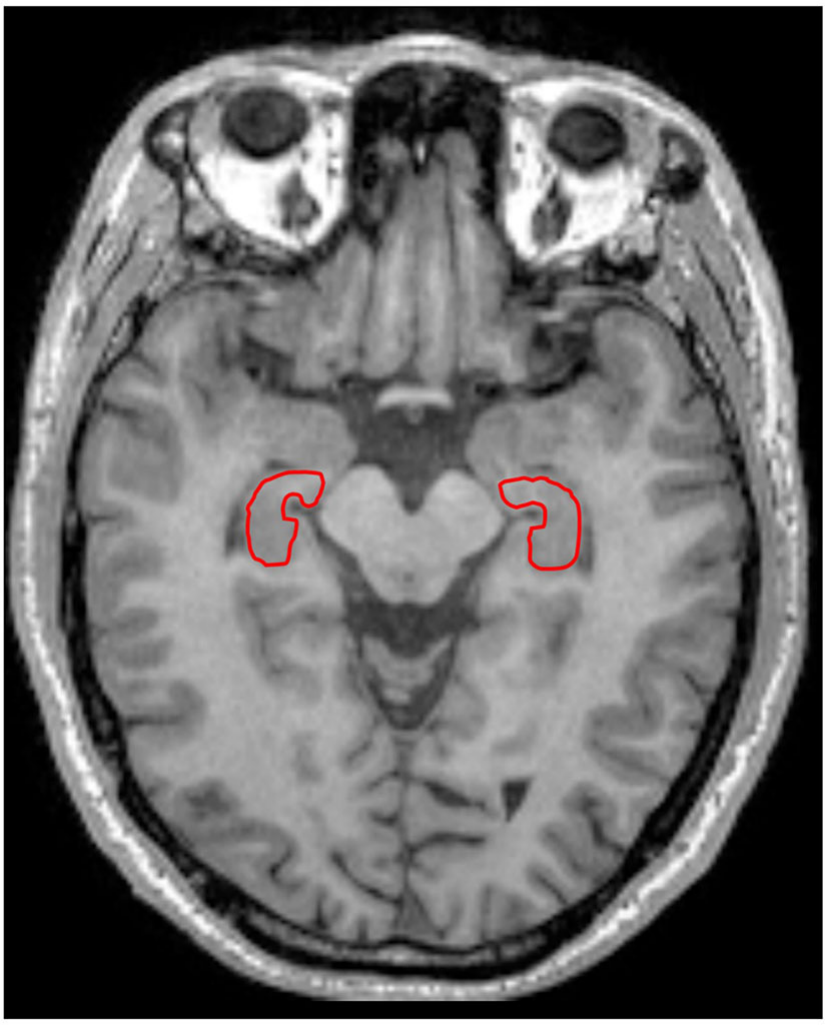
A 67-year-old male healthy control. The left and right hippocampus are outlined in red line in T1-weighted imaging.

In the present study, the hippocampus was chosen as the region of interest (ROI) (Figure 1). At first, the hippocampus was manually drawn slice by slice using MRICRON by a radiologist (LD, 5 years' working experience) on the T1 structure images, and then the drawn ROIs were registered onto lower-resolution dMRI, to more easily define the boundary of hippocampus. The ROIs' boundary was accurately segmented, and ambiguous voxels would be eliminated in all participants. Then the average values of α, *H*, ADC, generalized FA (gFA)(α), gFA(*H*), and gFA(ADC) in the left and right hippocampus were acquired.

### Image Analysis

First, the obtained images were corrected for head motion and eddy current distortions by FSL tools (Cha, 2006). In the dMRI acquisition, ADC maps were calculated using the images obtained at b-values of 0 and 954 s/mm^2^ (closest to conventional 1,000 s/mm^2^ b-value). We used the FM model to analyze the images. According to the FM-based dMRI theory (Sui et al., 2015), the following formula can be used to calculate diffusion-induced signal decay:

(1) $$\begin{array}{r} S/S_{0}=\exp\left(-\eta D_{\alpha ,H}\gamma^{\alpha }G_{0}^{\alpha }\Delta^{\alpha +\alpha H}\right) \end{array}$$

where *D*_α,*H*_ represents the diffusion coeffcient of anomalous diffusion and γ represents the gyromagnetic ratio. *G*_0_ represents the diffusion gradient amplitude, and Δ represents the gradient separation time. η is a dimensionless number, which can be calculated using α, *H*, δ, and Δ in the following formula (Xu et al., 2017b, 2018):

(2) $$\begin{array}{l} \eta =\frac{1}{{\left(1+\mu \right)}^{\alpha }}[\int_{0}^{\delta /\Delta }|{\left(\frac{\delta }{\Delta }+1-u\right)}^{1+\mu }-\;{\left(1-u\right)}^{1+\mu }\\ \;-{{\left(\frac{\delta }{\Delta }-u\right)}^{1+\mu }|}^{\alpha }du+\;\int_{\delta /\Delta }^{1}|{\left(\frac{\delta }{\Delta }+1-u\right)}^{1+\mu }\\ \;-{\left(1-u\right)}^{1+\mu }{|}^{\alpha }du+\;\int_{1}^{1+\delta /\Delta }{\left(\frac{\delta }{\Delta }+1-u\right)}^{\alpha +\alpha \mu }du] \end{array}$$

where μ = *H* − 1/α, and μ is the memory parameter. Along each direction, the signal attenuation at each voxel is fitted to **Equation 1** separately. We used the trust-region-reflective non-linear fitting algorithm in MATLAB (MathWorks, Natick, MA) to perform the fitting procedures.

A metric similar to FA, called gFA, is introduced to quantify anisotropy, where the sample standard deviation is divided by the root mean square [35]:

(3) $$\begin{array}{r} gFA\left(V\right)=\sqrt{\frac{N}{N-1}\frac{\sum_{i=1}^{N}{\left(V_{i}-\bar{V}\right)}^{2}}{\sum_{i=1}^{N}V_{i}^{2}}} \end{array}$$

where *N* represents the number of sampling directions, including three directions in this research, and *V* refers to the parameter values to be measured. $\bar{V}$ is the directionally averaged value, and *V*_*i*_ is the value in the *i*-th direction. The gFA maps of α, *H*, and ADC were calculated.

### Statistical Analysis

Among the mild AD group, moderate AD group, and healthy control group, gender was analyzed using the chi-square (χ^2^) test, and the age, education, and MMSE score were compared using one-way ANOVA. The MoCA score was compared using a two-sample *t*-test between the mild AD group and moderate AD group, since the MoCA score was not assessed in healthy control. Except for gender, the data were shown in the form of mean ± SD.

The gFA values of α, *H*, and ADC were compared using a one-way ANOVA test and *post-hoc* Tukey test among the healthy group, mild AD group, and moderate AD group. Moreover, receiver-operating characteristic (ROC) curves were performed to evaluate the diagnostic capability of each gFA value in differentiating AD patients from healthy controls and distinguishing mild AD patients from moderate AD patients by the area under the curve (AUC). Additionally, multivariate logistic regression analysis was utilized to assess the diagnostic performances of the combination of the anisotropy value and the directionally averaged value. For example, the probability of the combination of *H* and gFA(*H*) can be expressed as

(4) $$\begin{array}{r} P\left(\text{high}-\text{grade}|\left\{\text{gFA}\left(\alpha \right),\text{ gFA}\left(H\right)\right\}\right)\\ \;=\frac{\exp\left(a_{0}+a_{1}\text{gFA}\left(\alpha \right)+\;a_{2}\text{gFA}\left(H\right)\right)}{1+\exp\left(a_{0}+a_{1}\text{gFA}\left(\alpha \right)+\;a_{2}\text{gFA}\left(H\right)\right)} \end{array}$$

where *a*_1_ and *a*_2_ are the regression coeffcients for *H* and gFA(*H*), respectively, and *a*_0_ is a constant. The probabilities of other combinations can be expressed in a similar method. These probability values were used as the test variables in the ROC analysis.

In AD patients, the correlations between gFA(α), gFA(*H*), and gFA(ADC) values and the cognitive functions evaluated by MMSE and MoCA scales were investigated using Pearson correlation analysis. *P*-values < 0.05 were considered statistically significant.

## Results

### Characteristics of All Subjects

The demographic information and clinical cognition scores in all subjects are summarized in Table 1. Ultimately, 24 AD patients (69.0 ± 8.1 years) and 11 healthy controls (65.3 ± 6.6 years) were enrolled in this study. Then patients with AD were divided into two groups [the mild AD group (six males and six females, mean age 65.8 ± 10.1 years) and the moderate AD group (three males and nine females, mean age 72.1 ± 3.8 years)] according to their MMSE score and education level. The general division criteria are as follows: 21 ≤ MMSE score ≤ 25 was considered as mild AD, and 11 ≤ MMSE score ≤ 20 was considered as moderate AD (Perneczky et al., 2006). As demonstrated in Table 1, the age, gender, and education level of the three groups were matched (*P* > 0.05), while there was a significant difference in the MMSE score among the three groups (*P* < 0.05). MoCA score was significantly different between the mild AD group and moderate AD group, and a significant difference was found between the two groups (*P* < 0.05).

**Figure 2:**
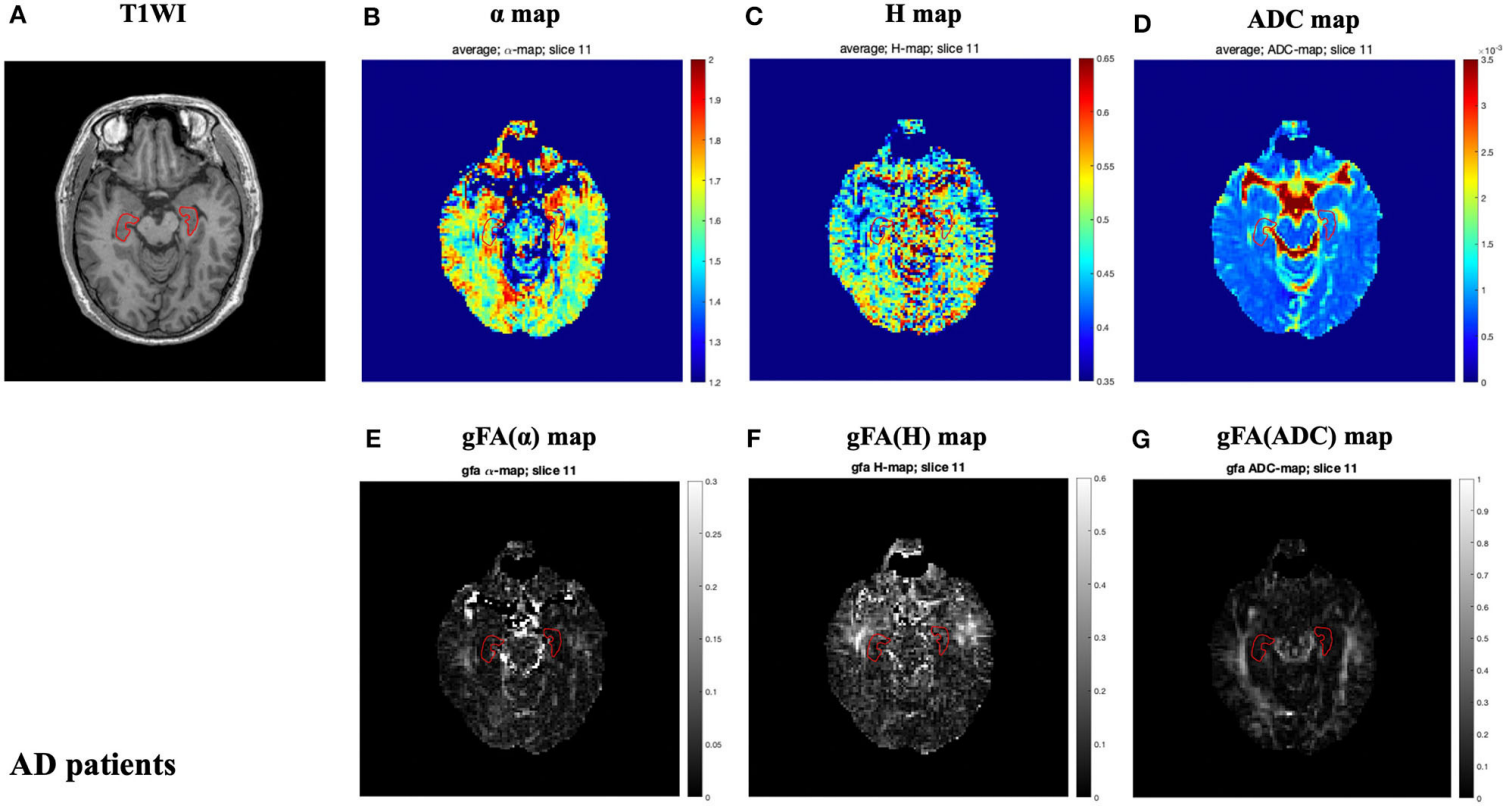
A 58-year-old male patient with AD. Top row: T1-weighed image **(A)** and directionally averaged maps of α, *H*, and ADC [**(B–D)**, respectively]. Bottom row: generalized fractional anisotropy (gFA) maps of α, *H*, and ADC [**(E–G)**, respectively]. The bilateral hippocampus is shown with red outlines in all maps. AD, Alzheimer's disease; ADC, apparent diffusion coefficient; gFA, generalized fractional anisotropy.

**Figure 3:**
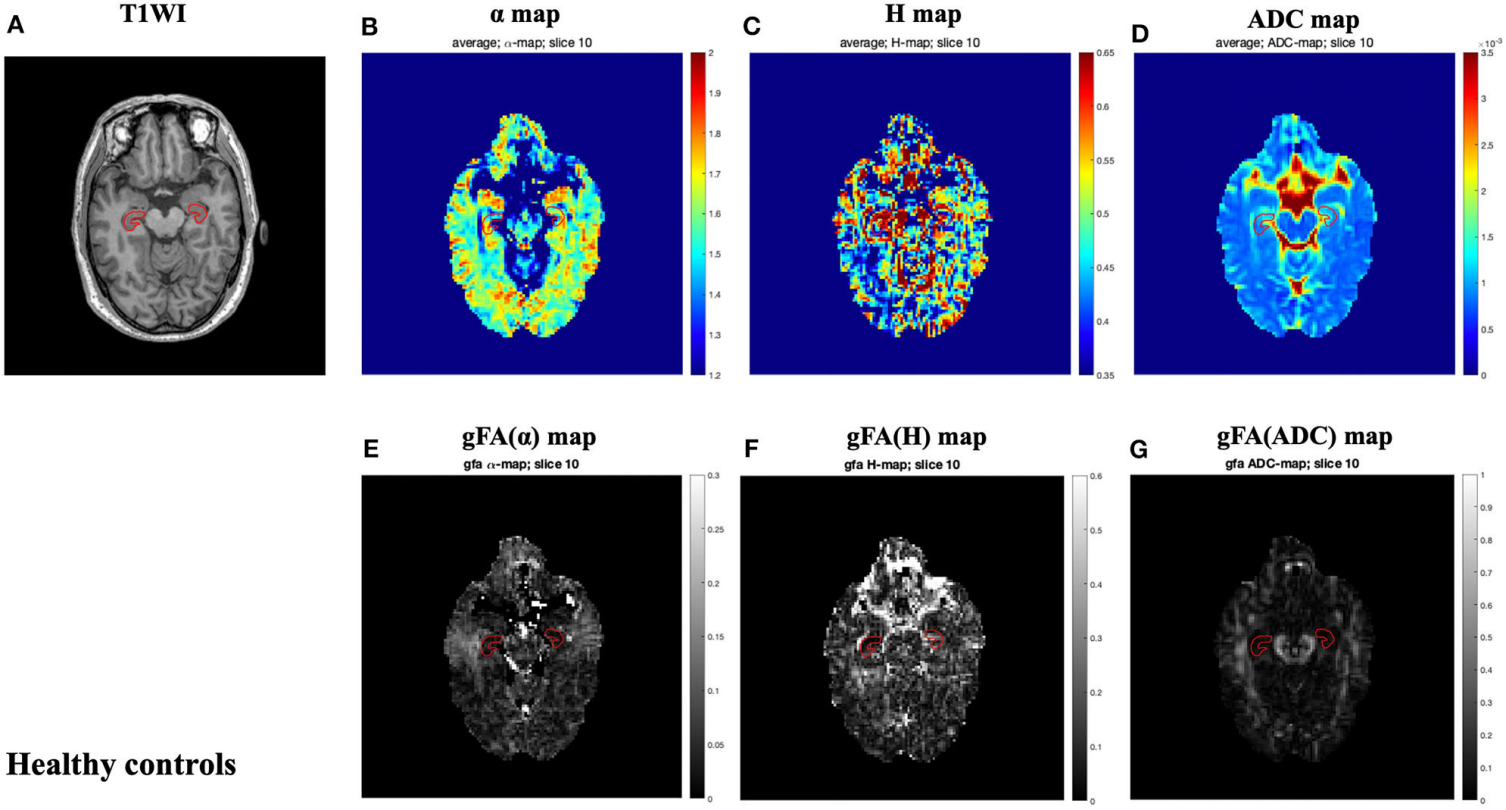
A 60-year-old male healthy control. Top row: axial T1-weighed image **(A)** and directionally averaged maps of α, *H*, and ADC [**(B–D)**, respectively]. Bottom row: gFA maps of α, *H*, and ADC [**(E–G)**, respectively]. The bilateral hippocampus is shown with red outlines in all maps. ADC, apparent diffusion coefficient; gFA, generalized fractional anisotropy.

The locations of the bilateral hippocampus in the T1-weighted image are shown in Figure 1. Figures 2, 3 show the representative maps of a 58-year-old male patient with AD and a 60-year-old male healthy patient, including the 3D T1-weighted images; directionally averaged maps of α, *H*, and ADC; and the gFA maps of α, *H*, and ADC. From Figures 2, 3, we found that there no outstanding contrasts between the hippocampus and other brain regions were observed by the naked eye.

### Comparisons of gFA(α), gFA(*H*), and gFA(ADC) Values Among Three Groups

**Figure 4:**
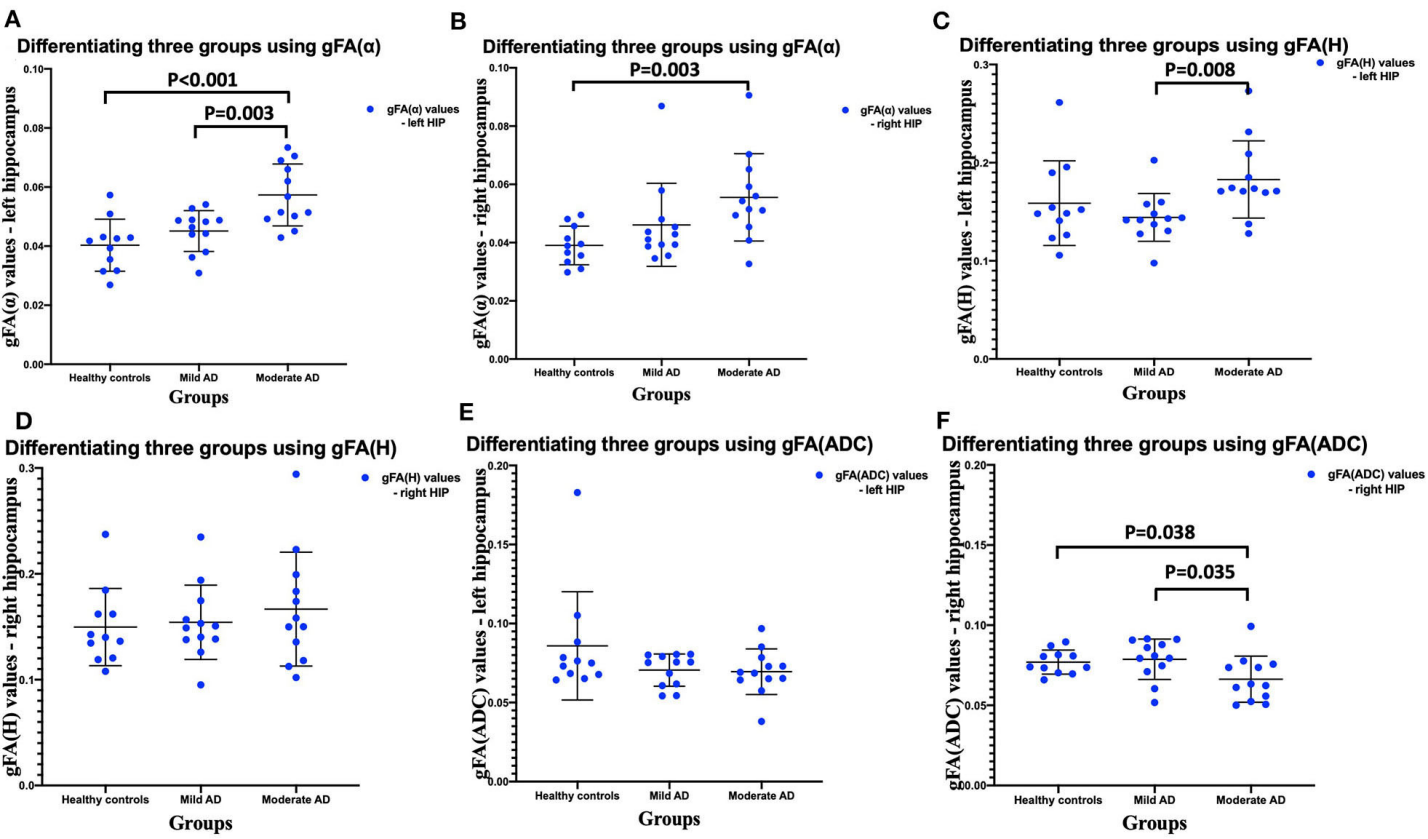
Comparisons among three groups: healthy control group, mild AD patient group, and moderate AD patient group **(A–F)**. Scatter plots show that gFA(α) and gFA(*H*) of the left hippocampus **(A,C)**, and gFA(ADC) values of the right hippocampus **(F)** can readily separate the mild AD patients and moderate AD patients, and gFA(α) of the bilateral hippocampus **(A,B)**, gFA(ADC) values of right hippocampus **(F)** can easily distinguish the moderate AD patients and healthy controls. *n* = 12 for mild AD patients, *n* = 12 for moderate AD patients, and *n* = 11 for healthy controls. *P* < 0.05 was considered as significant. AD, Alzheimer's disease; gFA, generalized fractional anisotropy; ADC, apparent diffusion coefficient.

The gFA values of α, *H*, and ADC of the left and right hippocampus in all participants are summarized in Table 2. Data are presented in the form of mean ± SD. The comparisons among three groups in gFA(α), gFA(*H*), and gFA(ADC) are shown in Figure 4. From Figure 4, we found that the gFA(α) and gFA(*H*) values of the moderate AD group were higher than those of the mild AD group (*P* = 0.003, *P* = 0.008, separately) in the left ROI (Figures 4A,C). We also found that the gFA(α) values of the moderate AD group were higher than those of the healthy control group (*P* < 0.001, *P* = 0.003, separately, Figures 4A,B) in the bilateral ROI, and the gFA(ADC) values of the moderate AD group were lower than those of the healthy control group and mild AD group in the right ROI (*P* = 0.038, *P* = 0.035, separately, Figure 4F). No significant differences were found between the healthy control group and mild AD group (*P* > 0.05 for all, Figures 4A–F).

**Table 2:** Mean and SD of the gFA(α), gFA(*H*), and gFA(ADC) values of left and right hippocampus in all participants.

**Subjects**	**No**.	**ROIs**	**gFA(α)**	**gFA(H)**	**gFA(ADC)**
Controls	11	Left-hippocampus	0.0403 ± 0.0088	0.1588 ± 0.0431	0.0859 ± 0.0340
		Right-hippocampus	0.0390 ± 0.0066	0.1497 ± 0.0364	0.0770 ± 0.0075
Mild AD	12	Left-hippocampus	0.0451 ± 0.0069	0.1443 ± 0.0244	0.0705 ± 0.0102
		Right-hippocampus	0.0461 ± 0.0143	0.1542 ± 0.0351	0.0787 ± 0.0126
Moderate AD	12	Left-hippocampus	0.0573 ± 0.0105	0.1829 ± 0.0393	0.0695 ± 0.0144
		Right-hippocampus	0.0555 ± 0.0150	0.1667 ± 0.0539	0.0663 ± 0.0144

*SD, standard deviation; gFA, generalized fractional anisotropy; ADC, apparent diffusion coefficient; AD, Alzheimer's disease; ROI, region of interest*.

**Figure 5:**
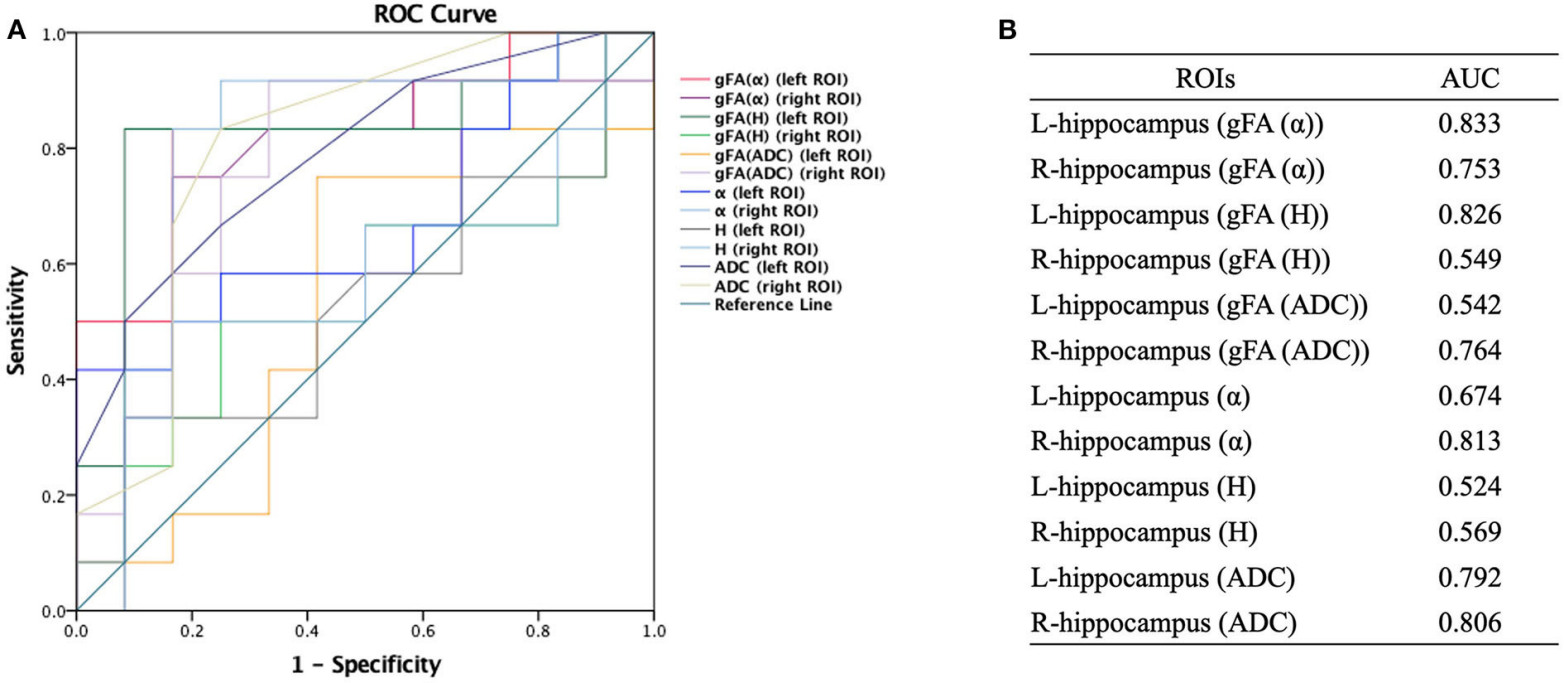
Receiver-operating characteristic (ROC) curve was generated using the individual gFA values and directionally averaged values of α, *H*, and ADC for differentiating mild and moderate AD patients (*n* = 12 in each group). **(A,B)** The area under the curve (AUC) of gFA(α) and gFA(*H*) values of the left hippocampus (AUC = 0.833, AUC = 0.826, separately) were larger than those of others. ROC, receiver operating characteristic; ROI, region of interest; gFA, generalized fractional anisotropy; ADC, apparent diffusion coefficient; AD, Alzheimer's disease; AUC, area under the curve.

**Figure 6:**
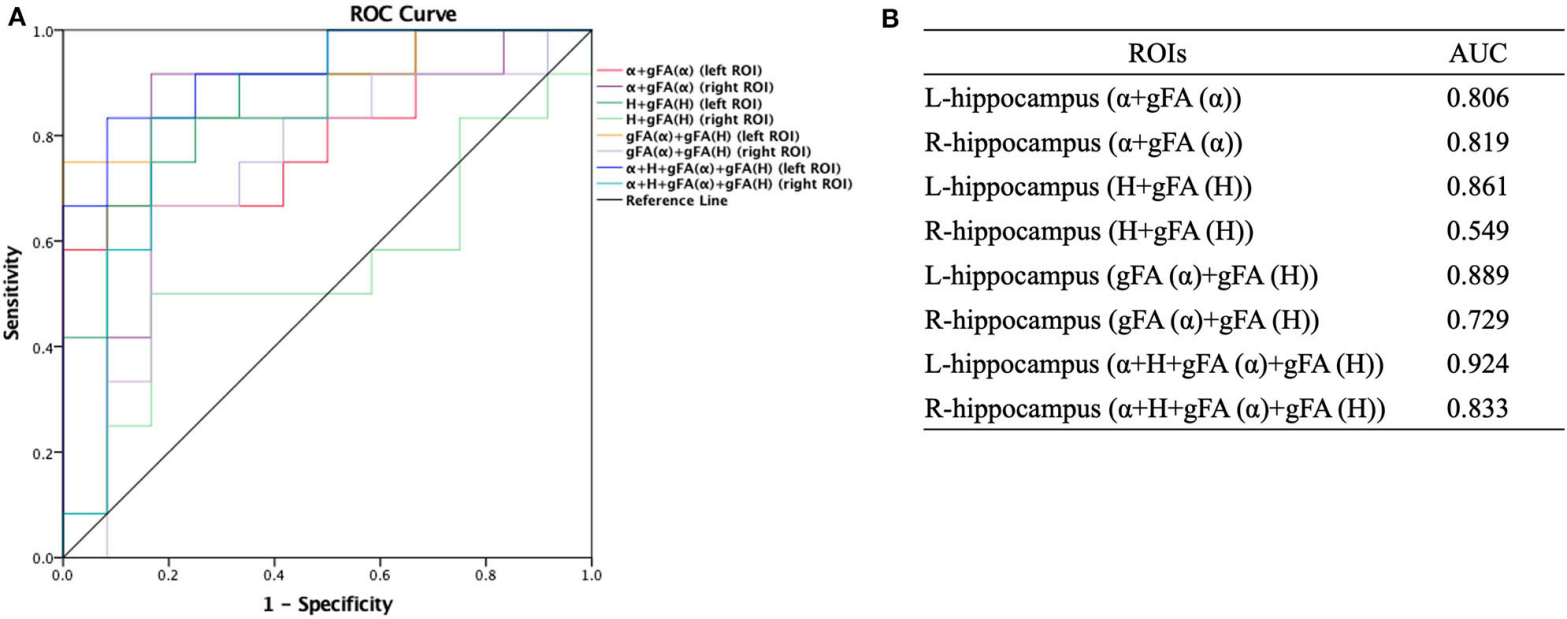
Receiver-operating characteristic (ROC) curve was generated using the combination of gFA values and averaged values of α, *H*, and ADC for differentiating mild and moderate AD patients (*n* = 12 in each group). **(A,B)** The combinations {α, gFA(α)} and {α, gFA(α), *H*, gFA(*H*)} of the bilateral ROI and {*H*, gFA(*H*)} and {gF(α) + gFA(*H*)} of the left ROI perfectly differentiate mild AD and moderate AD patients. The α combination {α, gFA(α)} and the *H* combination {*H*, gFA(*H*)} outperformed the directionally averaged α and *H*, respectively. ROC, receiver operating characteristic; ROI, region of interest; AUC, area under the curve; gFA, generalized fractional anisotropy; ADC, apparent diffusion coefficient; AD, Alzheimer's disease.

The performances in differentiating mild AD and moderate AD were illustrated by ROC analysis. Figure 5 depicts the ROC curves calculated from individual gFA values and directionally averaged maps of α, *H*, and ADC. Figure 5 shows that gFA(α) (AUC = 0.833) and gFA(*H*) (AUC = 0.826) of the left ROI and gFA(ADC) (AUC = 0.764) of the right ROI exhibited good capacity to differentiate the two groups. The other anisotropy measures of gFA parameters did not perform well. Figure 6 demonstrates the ROC curves calculated by combinations of different parameters, and some positive results were elucidated. More specifically, {α, gFA(α)} and {α, gFA(α), *H*, gFA(*H*)} of the bilateral ROI and {*H*, gFA(*H*)} and {gF(α) + gFA(*H*)} of the left ROI showed inspiring potencies in differentiating mild AD and moderate AD. It was noteworthy that, by combining the anisotropy information, the α combination {α, gFA(α)} (AUC = 0.806, left ROI; AUC = 0.819, right ROI) and the *H* combination {*H*, gFA(*H*)} (AUC = 0.861, left ROI; AUC = 0.549, right ROI) were significantly superior to the separate performances of the individual directionally averaged α (AUC = 0.674, left ROI; AUC = 813, right ROI) or *H* (AUC = 0.524, left ROI; AUC = 0.569, right ROI).

**Figure 7:**
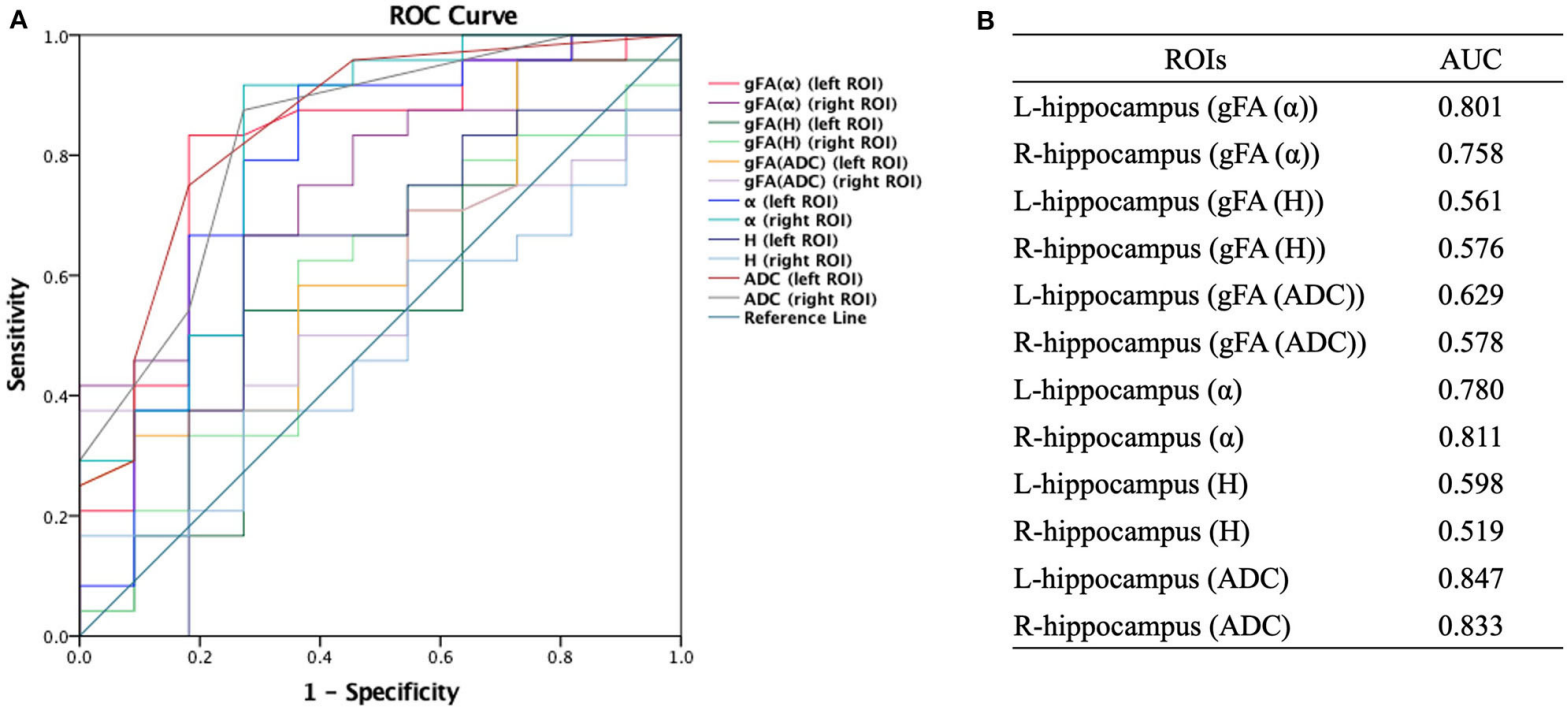
Receiver-operating characteristic (ROC) curve was generated using the individual gFA values and directionally averaged values of α, *H*, and ADC for differentiating healthy controls and AD patients. **(A,B)** The area under the curve (AUC) of gFA(α) values of the bilateral hippocampus (AUC = 0.801, AUC = 0.758, separately) was larger. ROC, receiver operating characteristic; ROI, region of interest; gFA, generalized fractional anisotropy; ADC, apparent diffusion coefficient; AD, Alzheimer's disease; AUC, area under the curve.

**Figure 8:**
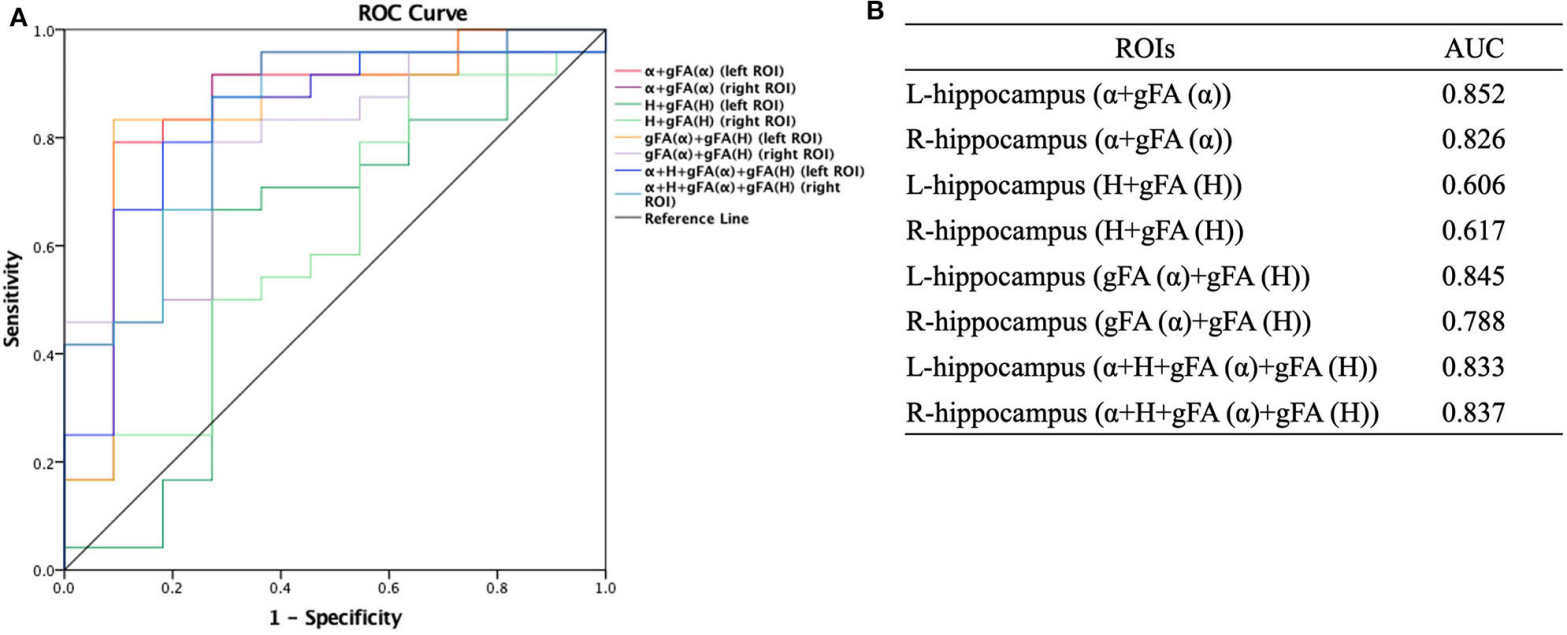
Receiver-operating characteristic (ROC) curve was generated using the combination of gFA values and averaged values of α, *H*, and ADC for differentiating AD patients and healthy controls (*n* = 24, *n* = 11, respectively). **(A,B)** The combinations {α, gFA(α)}, {gF(α) + gFA(*H*)}, and {α, gFA(α), *H*, gFA(*H*)} of the bilateral ROI perfectly separated AD patients and healthy controls. The α combination {α, gFA(α)} outperformed the directionally averaged α. ROC, receiver operating characteristic; ROI, region of interest; AUC, area under the curve; gFA, generalized fractional anisotropy; ADC, apparent diffusion coefficient; AD, Alzheimer's disease.

Similarly, ROC analysis in differentiating AD patients and healthy controls was also made. Figure 7 presents the ROC curves calculated from the individual gFA values and directionally averaged maps of α, *H*, and ADC for differentiating AD patients and healthy controls. As depicted in Figure 7, gFA(α) (AUC = 0.801, left ROI; AUC = 0.758, right ROI) values of the bilateral ROI exhibited a good capacity to differentiate the two groups. The anisotropy measures of gFA(*H*) and gFA(ADC) did not perform well. Figure 8 shows the ROC curves calculated from the combinations of different parameters, and the results validated some significant findings. Specifically, {α, gFA(α)}, {gF(α) + gFA(*H*)}, and {α, gFA(α), *H*, gFA(*H*)} of the bilateral ROI can perfectly separate AD patients and healthy controls. By combining the anisotropy information, the α combination {α, gFA(α)} (AUC = 0.852, left ROI; AUC = 0.826, right ROI) outperformed the individual directionally averaged α (AUC = 0.780, left ROI; AUC = 0.811, right ROI).

### Correlations Between Fractional Motion-Related Parameters and Mini-Mental State Examination Scores and Montreal Cognitive Assessment Scores

**Figure 9:**
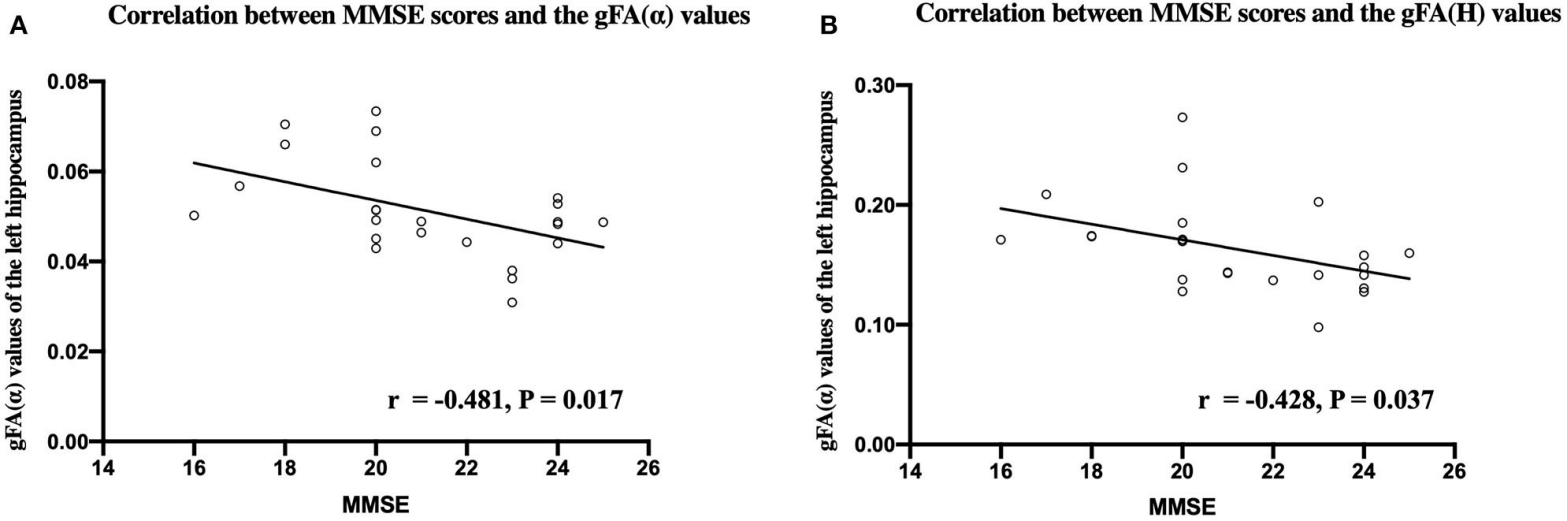
Correlations between MMSE scores and the gFA(α) **(A)** and gFA(*H*) **(B)** values of the left hippocampus in patients with AD. Pearson correlation was conducted. *n* = 24. MMSE, mini-mental state examination; gFA, generalized fractional anisotropy; AD, Alzheimer's disease.

Figure 9 shows that the gFA(α) and gFA(*H*) values of the left hippocampus were negatively correlated to corresponding MMSE score (*P* = 0.017, *P* = 0.037, respectively) in patients with AD. However, the correlations were not so strong, and there was no significant correlation after false discovery rate (FDR) correction and family-wise error rate (FWER) correction. Moreover, no significant correlations were found in other gFA parameters.

## Discussion

In the current study, we investigated the feasibility and effectiveness of the anisotropy of anomalous diffusion assessed by the FM model to differentiate and grade AD patients. We introduced a new anisotropy metric called gFA, and we explored its potencies in identifying AD patients and healthy controls and distinguishing mild AD and moderate AD patients. Our results demonstrated that the anisotropy of α and *H* of the left ROI exhibited good performances to grade AD patients, and the anisotropy of α of the bilateral ROI possessed good potencies to differentiate AD patients and healthy controls. It was worth noting that the diagnostic accuracy was increased when combined with the anisotropy metric with the commonly used directionally averaged value, indicating that the anisotropy metric could improve the diagnostic performances of directionally averaged values in identifying and grading AD patients.

An important finding in the present study was that the anisotropy of α and *H* showed significant superiority to distinguish mild AD and moderate AD patients and identify AD patients from healthy controls, in particular the α. In combination with the results of our previous study (Du et al., 2020), we reached a conclusion that both directionally averaged value and the anisotropy value of α exhibited excellent capacity to identify and grade AD patients, which indicated that α-related values may possess a specific advantage in the diagnosis and grading of AD. In line with the currently available data, α-related diffusion values probably already provide sufficient information regarding the differentiation and classification of AD, which is beneficial to shorten the scan time, simplify the test procedure, and improve medical efficiency. However, it should be validated by future studies with larger sample size.

Possible explanations for the diagnostic performance of gFA(α) are as follows. The symbol α describes the fluctuations of the random process. Excessive deposition of Aβ protein and neurofibrillary tangles in brain tissue are two main pathological changes in AD patients and can lead to neuronal apoptosis (Wang et al., 2015). These pathological alterations that emerged along with AD progression can result in subsequent neuronal apoptosis and encephalatrophy, which eventually reduce the volume of affected brain regions (especially the hippocampus). Moreover, it is well acknowledged that the α values depend on the structural complexity of the brain regions. The non-Gaussian water molecule diffusion is more active in a more complex brain region, and accordingly, the measured α values would be higher and gFA(α) values would be lower. Consistently, the degenerative pathological alterations (neuronal apoptosis and encephalatrophy) that occurred in AD progression can markedly decrease the structural complexity of the hippocampus (Jensen et al., 2005; Grinberg et al., 2011; Yoshida et al., 2013) and can manifest as different α values and gFA(α) values among healthy controls, mild AD, and moderate AD patients, which were observed in the current study.

Compared with other commonly used traditional techniques, such as DTI, using the FM model to calculate anisotropy of anomalous diffusion possesses several potential advantages. On the one hand, DTI quantifies the diffusion process of water molecule using a mono-exponential form. However, the observed dMRI signal decay curve in the brain deviates from the mono-exponential form. In this regard, the FM model was introduced and showed a better agreement between the measured signal decay curve and the fitted curves (Magin et al., 2008). On the other hand, the detected diffusion-time dependence of the MR signal shows a non-Gaussian nature of diffusion, while the DTI model assumes the molecular displacement in brain tissues with a 3D Gaussian ellipsoid (Fieremans et al., 2016). This defect was circumvented by the FM model.

The feasibility and effectiveness of anisotropy calculated by the FM model have been verified in this research. But potential clinical applications are not limited to the FM model. As shown in **Equation 1**, the α is an exponent of the diffusion gradient, which is proportional to the parameters of other dMRI models, like the stretching parameter γ in the stretched-exponential model (Bennett et al., 2003; Hall and Barrick, 2008; Zhou et al., 2010). Therefore, the clinical feasibility of anisotropy may be generalized to the α-like parameters of other models because of the intrinsic consistency of their anisotropic properties (Xu et al., 2017a).

The current results indicated that the gFA(α) and gFA(*H*) values of the left hippocampus were negatively correlated with MMSE score in patients with AD. This finding was partially consistent with the results of our previous study using directionally averaged values (Du et al., 2020), which further provided evidence for the reliability and repeatability of our findings. However, the correlations were not so strong and even not significant after FDR and FWER correction. This may be explained by the following reasons. In addition to the hippocampus, pathological alterations of AD affected other brain regions such as the prefrontal cortex and basal ganglion region, which synergistically contributed to cognitive impairments in AD patients. So there might be no linear relationship between cognitive scores and anisotropic values in the hippocampus. Moreover, the small sample size of this study may also affect the results, and further researches with larger sample size are needed to confirm these findings.

The limitations of the present study must be acknowledged. First, this is a single-institution study with a limited number of healthy controls and AD patients, and the results should be validated by further study with larger samples. We hope the independent validation of our results can be done at separated institutions. Second, the voxel of imaging is large, and a single voxel displays an average measurement of neuronal environment, which may decrease the sensitivity for brain tissue components occupying a small part of a voxel. Third, the x-, y-, and z-axes are the only three directions being applied by the diffusion gradients, and the accuracy of this study may be affected, as increased sampling directions are conducive to measuring the anisotropy of anomalous diffusion.

## Conclusions

In summary, the anisotropy of anomalous diffusion was successfully applied in differentiating and grading patients with AD. It was worth noting that the diagnostic performance was improved when the anisotropy metric was combined with commonly used directionally averaged value in identifying and grading AD patients. The anisotropy of anomalous diffusion calculated by the FM model may provide novel insights to profoundly elucidate the neuropathology process of AD.

## Data Availability Statement

The original contributions presented in the study are included in the article/Supplementary Materials, further inquiries can be directed to the corresponding author.

## Ethics Statement

The studies involving human participants were reviewed and approved by China-Japan Friendship Hospital. The participants provided their written informed consent to participate in this study. Written informed consent was obtained from the individuals for the publication of any potentially identifiable images or data included in this article.

## Author Contributions

LD, ZZ, and BX designed this research, analyzed the MRI data, and drafted this manuscript. SS, WG, XL, YC, YW, JL, and BL did the MRI scanning. BX and JG wrote and revised the content of the FM model theory, separately. GM revised the whole manuscript. All authors approved the final manuscript.

## Conflict of Interest

BX was employed by Beijing Intelligent Brain Cloud Inc company. The remaining authors declare that the research was conducted in the absence of any commercial or financial relationships that could be construed as a potential conflict of interest.

## References

[B1] Alzheimer's, Association. (2016). 2016 Alzheimer's disease facts and figures. Alzheimers Dement 12, 459–509. 10.1016/j.jalz.2016.03.001 27570871

[B2] Anckaerts C. Blockx I. Summer P. Michael J. Hamaide J. Kreutzer C.. (2019). Early functional connectivity deficits and progressive microstructural alterations in the TgF344-AD rat model of Alzheimer's Disease: a longitudinal MRI study. Neurobiol. Dis. 124, 93–107. 10.1016/j.nbd.2018.11.010 30445024

[B3] Basser P. J. Mattiello J. LeBihan D. (1994a). Estimation of the effective self-diffusion tensor from the NMR spin echo. J. Magn. Reson. B 103, 247–254. 10.1006/jmrb.1994.1037 8019776

[B4] Basser P. J. Mattiello J. LeBihan D. (1994b). MR diffusion tensor spectroscopy and imaging. Biophys. J. 66, 259–267. 10.1016/S0006-3495(94)80775-1 8130344 PMC1275686

[B5] Beaulieu C. (2002). The basis of anisotropic water diffusion in the nervous system - a technical review. NMR Biomed. 15, 435–455. 10.1002/nbm.782 12489094

[B6] Bennett K. M. Schmainda K. M. Bennett R. T. Rowe D. B. Lu H. Hyde J. S. (2003). Characterization of continuously distributed cortical water diffusion rates with a stretched-exponential model. Magn. Reson. Med. 50, 727–734. 10.1002/mrm.10581 14523958

[B7] Bergamino M. Nespodzany A. Baxter L. C. Burke A. Caselli R. J. Sabbagh M. N. (2020). Preliminary assessment of intravoxel incoherent motion diffusion-weighted MRI (IVIM-DWI) metrics in Alzheimer's disease. J. Magn. Reson. Imaging 4:e27272 10.1002/jmri.27272 32621405

[B8] Bouts M. Moller C. Hafkemeijer A. van Swieten J. C. Dopper E. van der Flier W. M.. (2018). Single subject classification of alzheimer's disease and behavioral variant frontotemporal dementia using anatomical, diffusion tensor, and resting-state functional magnetic resonance imaging. J. Alzheimers Dis. 62, 1827–1839. 10.3233/JAD-170893 29614652

[B9] Braak H. Braak E. (1991). Neuropathological stageing of Alzheimer-related changes. Acta Neuropathol. 82, 239–259. 10.1007/bf00308809 1759558

[B10] Brueggen K. Dyrba M. Cardenas-Blanco A. Schneider A. Fliessbach K. Buerger K.. (2019). Structural integrity in subjective cognitive decline, mild cognitive impairment and Alzheimer's disease based on multicenter diffusion tensor imaging. J. Neurol. 266, 2465–2474. 10.1007/s00415-019-09429-3 31227891

[B11] Burnecki K. Weron A. (2010). Fractional Levy stable motion can model subdiffusive dynamics. Phys. Rev. E Stat. Nonlin. Soft Matter Phys. 82:021130. 10.1103/PhysRevE.82.021130 20866798

[B12] Cha S. (2006). Update on brain tumor imaging: from anatomy to physiology. AJNR Am. J. Neuroradiol. 27, 475–487. 16551981 PMC7976984

[B13] Cummings J. (2017). Disease modification and neuroprotection in neurodegenerative disorders. Transl. Neurodegener. 6:25. 10.1186/s40035-017-0096-2 29021896 PMC5613313

[B14] De Santis S. Gabrielli A. Bozzali M. Maraviglia B. Macaluso E. Capuani S. (2011). Anisotropic anomalous diffusion assessed in the human brain by scalar invariant indices. Magn. Reson. Med. 65, 1043–1052. 10.1002/mrm.22689 21413068

[B15] Du L. Xu B. Zhao Z. Han X. Gao W. Shi S.. (2020). Identification and classification of Alzheimer's disease patients using novel fractional motion model. Front. Neurosci. 14:767. 10.3389/fnins.2020.00767 33071719 PMC7533574

[B16] Dyrba M. Barkhof F. Fellgiebel A. Filippi M. Hausner L. Hauenstein K.. (2015). Predicting prodromal Alzheimer's disease in subjects with mild cognitive impairment using machine learning classification of multimodal multicenter diffusion-tensor and magnetic resonance imaging data. J. Neuroimaging 25, 738–747. 10.1111/jon.12214 25644739

[B17] Fieremans E. Burcaw L. M. Lee H. H. Lemberskiy G. Veraart J. Novikov D. S. (2016). *In vivo* observation and biophysical interpretation of time-dependent diffusion in human white matter. Neuroimage 129, 414–427. 10.1016/j.neuroimage.2016.01.018 26804782 PMC4803645

[B18] Finsterwalder S. Vlegels N. Gesierich B. Araque Caballero M. A. Weaver N. A. Franzmeier N.. (2020). Small vessel disease more than Alzheimer's disease determines diffusion MRI alterations in memory clinic patients. Alzheimers Dement. 18. 10.1002/alz.12150 32808747 PMC8102202

[B19] Folstein M. F. Folstein S. E. McHugh P. R. (1975). “Mini-mental state”. A practical method for grading the cognitive state of patients for the clinician. J. Psychiatr. Res. 12, 189–198. 10.1016/0022-3956(75)90026-6 1202204

[B20] Grinberg F. Farrher E. Kaffanke J. Oros-Peusquens A. M. Shah N. J. (2011). Non-Gaussian diffusion in human brain tissue at high b-factors as examined by a combined diffusion kurtosis and biexponential diffusion tensor analysis. Neuroimage 57, 1087–1102. 10.1016/j.neuroimage.2011.04.050 21596141

[B21] Hall M. G. Barrick T. R. (2008). From diffusion-weighted MRI to anomalous diffusion imaging. Magn. Reson. Med. 59, 447–455. 10.1002/mrm.21453 18224695

[B22] Hall M. G. Barrick T. R. (2012). Two-step anomalous diffusion tensor imaging. NMR Biomed. 25, 286–294. 10.1002/nbm.1747 21812048

[B23] Harrison J. R. Bhatia S. Tan Z. X. Mirza-Davies A. Benkert H. Tax C. M. W.. (2020). Imaging Alzheimer's genetic risk using diffusion MRI: a systematic review. Neuroimage Clin. 27:102359. 10.1016/j.nicl.2020.102359 32758801 PMC7399253

[B24] Hong Y. J. Yoon B. Lim S. C. Shim Y. S. Kim J. Y. Ahn K. J.. (2013). Microstructural changes in the hippocampus and posterior cingulate in mild cognitive impairment and Alzheimer's disease: a diffusion tensor imaging study. Neurol. Sci. 34, 1215–1221. 10.1007/s10072-012-1225-4 23109096

[B25] Hyman B. T. Van Hoesen G. W. Damasio A. R. Barnes C. L. (1984). Alzheimer's disease: cell-specific pathology isolates the hippocampal formation. Science 225, 1168–1170. 10.1126/science.6474172 6474172

[B26] Ingo C. Magin R. L. Colon-Perez L. Triplett W. Mareci T. H. (2014). On random walks and entropy in diffusion-weighted magnetic resonance imaging studies of neural tissue. Magn. Reson. Med. 71, 617–627. 10.1002/mrm.24706 23508765 PMC4930657

[B27] Jensen J. H. Helpern J. A. Ramani A. Lu H. Kaczynski K. (2005). Diffusional kurtosis imaging: the quantification of non-gaussian water diffusion by means of magnetic resonance imaging. Magn. Reson. Med. 53, 1432–1440. 10.1002/mrm.20508 15906300

[B28] Karaman M. M. Sui Y. Wang H. Magin R. L. Li Y. Zhou X. J. (2016). Differentiating low- and high-grade pediatric brain tumors using a continuous-time random-walk diffusion model at high b-values. Magn. Reson. Med. 76, 1149–1157. 10.1002/mrm.26012 26519663 PMC4852163

[B29] Khan A. Corbett A. Ballard C. (2017). Emerging treatments for Alzheimer's disease for non-amyloid and non-tau targets. Expert Rev. Neurother. 17, 683–695. 10.1080/14737175.2017.1326818 28490260

[B30] Kidd M. (1963). Paired helical filaments in electron microscopy of Alzheimer's disease. Nature 197, 192–193. 10.1038/197192b0 14032480

[B31] Kwee T. C. Galban C. J. Tsien C. Junck L. Sundgren P. C. Ivancevic M. K.. (2010a). Comparison of apparent diffusion coefficients and distributed diffusion coefficients in high-grade gliomas. J. Magn. Reson. Imaging 31, 531–537. 10.1002/jmri.22070 20187193 PMC2918396

[B32] Kwee T. C. Galban C. J. Tsien C. Junck L. Sundgren P. C. Ivancevic M. K.. (2010b). Intravoxel water diffusion heterogeneity imaging of human high-grade gliomas. NMR Biomed. 23, 179–187. 10.1002/nbm.1441 19777501 PMC4123199

[B33] La Rocca M. Amoroso N. Monaco A. Bellotti R. Tangaro S. (2018). A novel approach to brain connectivity reveals early structural changes in Alzheimer's disease. Physiol. Meas. 39:074005. 10.1088/1361-6579/aacf1f 29943735

[B34] Le Bihan D. (1995). Molecular diffusion, tissue microdynamics and microstructure. NMR Biomed. 8, 375–386. 10.1002/nbm.1940080711 8739274

[B35] Le Bihan D. Johansen-Berg H. (2012). Diffusion MRI at 25: exploring brain tissue structure and function. Neuroimage 61, 324–341. 10.1016/j.neuroimage.2011.11.006 22120012 PMC3683822

[B36] Lee P. Ryoo H. Park J. Jeong Y. Alzheimer's Disease Neuroimaging Initiative. (2017). Morphological and microstructural changes of the hippocampus in early MCI: a study utilizing the alzheimer's disease neuroimaging initiative database. J. Clin. Neurol. 13, 144–154. 10.3988/jcn.2017.13.2.144 28176504 PMC5392456

[B37] Magdziarz M. Weron A. Burnecki K. Klafter J. (2009). Fractional brownian motion versus the continuous-time random walk: a simple test for subdiffusive dynamics. Phys. Rev. Lett. 103:180602. 10.1103/PhysRevLett.103.180602 19905793

[B38] Magin R. L. Abdullah O. Baleanu D. Zhou X. J. (2008). Anomalous diffusion expressed through fractional order differential operators in the Bloch-Torrey equation. J. Magn. Reson. 190, 255–270. 10.1016/j.jmr.2007.11.007 18065249

[B39] Marcos Dolado A. Gomez-Fernandez C. Yus Fuertes M. Barabash Bustelo A. Marcos-Arribas L. Lopez-Mico C.. (2019). Diffusion tensor imaging measures of brain connectivity for the early diagnosis of Alzheimer's disease. Brain Connect 9, 594–603. 10.1089/brain.2018.0635 31244329

[B40] Mattsson N. Insel P. S. Donohue M. Jogi J. Ossenkoppele R. Olsson T.. (2019). Predicting diagnosis and cognition with (18)F-AV-1451 tau PET and structural MRI in Alzheimer's disease. Alzheimers Dement 15, 570–580. 10.1016/j.jalz.2018.12.001 30639421

[B41] Mayo C. D. Mazerolle E. L. Ritchie L. Fisk J. D. Gawryluk J. R. Alzheimer's Disease Neuroimaging Initiative. (2017). Longitudinal changes in microstructural white matter metrics in Alzheimer's disease. Neuroimage Clin. 13, 330–338. 10.1016/j.nicl.2016.12.012 28066707 PMC5200876

[B42] McKhann G. Drachman D. Folstein M. Katzman R. Price D. Stadlan E. M. (1984). Clinical diagnosis of Alzheimer's disease: report of the NINCDS-ADRDA work group under the auspices of department of health and human services task force on Alzheimer's disease. Neurology 34, 939–944. 10.1212/wnl.34.7.939 6610841

[B43] Mulkern R. V. Gudbjartsson H. Westin C. F. Zengingonul H. P. Gartner W. Guttmann C. R.. (1999). Multi-component apparent diffusion coefficients in human brain. NMR Biomed. 12, 51–62. 10.1002/(sici)1099-1492(199902)12:1<51::aid-nbm546>3.0.co;2-e 10195330

[B44] Perneczky R. Wagenpfeil S. Komossa K. Grimmer T. Diehl J. Kurz A. (2006). Mapping scores onto stages: mini-mental state examination and clinical dementia rating. Am. J. Geriatr. Psychiatry 14, 139–144. 10.1097/01.JGP.0000192478.82189.a8 16473978

[B45] Reddy P. H. Oliver D. M. (2019). Amyloid beta and phosphorylated tau-induced defective autophagy and mitophagy in Alzheimer's disease. Cells 8:488. 10.3390/cells8050488 31121890 PMC6562604

[B46] Schouten T. M. Koini M. Vos F. Seiler S. Rooij M. Lechner A.. (2017). Individual classification of Alzheimer's disease with diffusion magnetic resonance imaging. Neuroimage 152, 476–481. 10.1016/j.neuroimage.2017.03.025 28315741

[B47] Sui Y. Wang H. Liu G. Damen F. W. Wanamaker C. Li Y.. (2015). Differentiation of low- and high-grade pediatric brain tumors with high b-value diffusion-weighted MR imaging and a fractional order calculus model. Radiology 277, 489–496. 10.1148/radiol.2015142156 26035586 PMC4627432

[B48] Takahashi H. Ishii K. Kashiwagi N. Watanabe Y. Tanaka H. Murakami T.. (2017). Clinical application of apparent diffusion coefficient mapping in voxel-based morphometry in the diagnosis of Alzheimer's disease. Clin. Radiol. 72, 108–115. 10.1016/j.crad.2016.11.002 27908444

[B49] Tang X. Qin Y. Wu J. Zhang M. Zhu W. Miller M. I. (2016). Shape and diffusion tensor imaging based integrative analysis of the hippocampus and the amygdala in Alzheimer's disease. Magn. Reson. Imaging 34, 1087–1099. 10.1016/j.mri.2016.05.001 27211255 PMC6058972

[B50] Tchalla A. E. Clement J. P. Saulnier I. Beaumatin B. Lachal F. Gayot C.. (2018). Predictors of rapid cognitive decline in patients with mild-to-moderate alzheimer disease: a prospective cohort study with 12-month follow-up performed in memory clinics. Dement Geriatr. Cogn. Disord. 45, 56–65. 10.1159/000487938 29684916

[B51] Thiessen J. D. Glazner K. A. Nafez S. Schellenberg A. E. Buist R. Martin M.. (2010). Histochemical visualization and diffusion MRI at 7 Tesla in the TgCRND8 transgenic model of Alzheimer's disease. Brain Struct. Funct. 215, 29–36. 10.1007/s00429-010-0271-z 20512361

[B52] Wang D. Guo Z. H. Liu X. H. Li Y. H. Wang H. (2015). Examination of hippocampal differences between Alzheimer disease, amnestic mild cognitive impairment and normal aging: diffusion kurtosis. Curr. Alzheimer Res. 12, 80–87. 10.2174/1567205012666141218142422 25523422

[B53] Wegmann S. Jung Y. J. Chinnathambi S. Mandelkow E. M. Mandelkow E. Muller D. J. (2010). Human Tau isoforms assemble into ribbon-like fibrils that display polymorphic structure and stability. J. Biol. Chem. 285, 27302–27313. 10.1074/jbc.M110.145318 20566652 PMC2930729

[B54] Weiss M. (2013). Single-particle tracking data reveal anticorrelated fractional Brownian motion in crowded fluids. Phys. Rev. E Stat. Nonlin. Soft Matter Phys. 88:010101. 10.1103/PhysRevE.88.010101 23944389

[B55] Wortmann M. (2012). Dementia: a global health priority - highlights from an ADI and World Health Organization report. Alzheimers Res. Ther. 4:40. 10.1186/alzrt143 22995353 PMC3580397

[B56] Xu B. Gong G. Fan Y. Wu B. Gao J. H. (2017a). Directional sensitivity of anomalous diffusion in human brain assessed by tensorial fractional motion model. Magn. Reson. Imaging 42, 74–81. 10.1016/j.mri.2017.05.006 28577902

[B57] Xu B. Su L. Wang Z. Fan Y. Gong G. Zhu W.. (2017b). Anomalous diffusion in cerebral glioma assessed using a fractional motion model. Magn. Reson. Med. 78, 1944–1949. 10.1002/mrm.26581 28054416

[B58] Xu B. Su L. Wang Z. Fan Y. Gong G. Zhu W.. (2018). Anisotropy of anomalous diffusion improves the accuracy of differentiating low- and high-grade cerebral gliomas. Magn. Reson. Imaging 51, 14–19. 10.1016/j.mri.2018.04.005 29673894

[B59] Xue Y. Zhang Z. Wen C. Liu H. Wang S. Li J.. (2019). Characterization of alzheimer's disease using ultra-high b-values apparent diffusion coefficient and diffusion kurtosis imaging. Aging Dis. 10, 1026–1036. 10.14336/AD.2018.1129 31595200 PMC6764724

[B60] Yablonskiy D. A. Bretthorst G. L. Ackerman J. J. (2003). Statistical model for diffusion attenuated MR signal. Magn. Reson. Med. 50, 664–669. 10.1002/mrm.10578 14523949 PMC2140254

[B61] Yoshida S. Oishi K. Faria A. V. Mori S. (2013). Diffusion tensor imaging of normal brain development. Pediatr. Radiol. 43, 15–27. 10.1007/s00247-012-2496-x 23288475 PMC3703661

[B62] Zhou X. J. Gao Q. Abdullah O. Magin R. L. (2010). Studies of anomalous diffusion in the human brain using fractional order calculus. Magn. Reson. Med. 63, 562–569. 10.1002/mrm.22285 20187164

